# Assessing awareness of colorectal cancer symptoms and screening in a peripheral colorectal surgical unit: a survey based study

**DOI:** 10.1186/1471-2482-13-20

**Published:** 2013-06-22

**Authors:** Terri P McVeigh, Aoife J Lowery, Ronan M Waldron, Akhtar Mahmood, Kevin Barry

**Affiliations:** 1 Department of Surgery, Mayo General Hospital, Castlebar, Mayo, Ireland

**Keywords:** Colorectal cancer, Screening, Colonoscopy

## Abstract

**Background:**

The National Screening Program for colorectal cancer is scheduled to commence in the near future. Previous studies on the topic of colorectal cancer and screening have highlighted paucity in public awareness of epidemiology, symptoms and signs of colorectal cancer. The aim of this study was to assess understanding of colorectal cancer and screening in a representative sample of the local catchment population of Mayo General Hospital.

**Methods:**

A prospective cohort study was instituted utilising an anonymous survey, which was distributed at consecutive general surgical out-patient clinics over a one month period prior to initiation of the screening program. Data collected included demographics, presenting complaint type and duration, and general knowledge of colorectal cancer facts. Attitudes towards screening were also evaluated.

**Results:**

Eighty-eight of the one hundred and thirty six patients sampled were female (65%). Thirty-six per cent of the sample was within the screening target age-group (55–74), with mean age 53years (+/−18). Most respondents recognised bleeding per rectum as a possible symptom of colorectal cancer. A significant proportion, however, incorrectly selected less sinister symptoms as concerning, while only fifty per cent correctly cited weight loss. Family history was acknowledged as a risk factor by fifty-seven per cent with age and gender cited less often (29%, 4%), while forty-seven per cent incorrectly cited stress as a risk. Screening was defined as testing of symptomatic patients or those with a positive family history by eighty-one per cent of respondents, with only nineteen per cent associating screening with an asymptomatic cohort. Strikingly, twenty-five per cent of patients would decline screening.

**Conclusions:**

There remains poverty of awareness regarding colorectal cancer. More public health initiatives are required to help improve understanding of the disease process, and to improve public compliance with the screening initiative.

## Background

Colorectal cancer in Ireland remains a significant health problem despite advances in treatment protocols. In 2011, 2270 newly diagnosed cases were documented, accounting for 12.9% of invasive cancers in the Irish population [1]. There were 910 colorectal cancer-related deaths in 2011, representing 11.1% of all cancer mortalities [1]. Compared to other European countries, both colorectal cancer incidence and mortality in Ireland were higher than the EU average [1]. It is indisputable therefore that colorectal cancer is an important health problem in Ireland. As a result of centralisation of cancer services, Ireland now has high-quality specialist centres offering multimodal treatments for this disease. Colorectal cancer is also one of the most intensively researched and well-understood pathologies, with a defined natural history. For this reason, colorectal cancer fulfils many of the disease-related WHO criteria for suitability of screening [2] but acceptability and suitability of screening tests remain to be determined.

The National Cancer Screening Service in Ireland plans to implement a national population-based colorectal cancer screening programme on a phased basis, starting with the population aged 60–69, with plans to include, in time, all patients aged 55-74 [3]. As part of this screening programme, willing participants aged 60–69 will be sent a screening Faecal Immunochemical Test kit every two years. An abnormal test will require an additional screening test in the form of a colonoscopy. For this phase of the screening programme, the National Cancer Screening Service has selected fifteen candidate screening colonoscopy units to undertake provision of screening colonoscopies. Mayo General Hospital, a secondary unit in the North-West of Ireland has been selected as one of these screening centres. This centre has 339 beds, and serves a predominantly rural-based population of 117000.

Previous work in our centre has highlighted a paucity of awareness of colorectal cancer symptoms in those patients presenting on an emergent basis with colorectal cancer [4]. Harewood et al. more recently published findings of similar lack of knowledge of this disease among a cohort attending a gastroenterology clinic in an urban-based teaching hospital [5]. This review also illustrated a suboptimal willingness of asymptomatic patients to undergo colonoscopy as a means of screening for colorectal cancer.

The aim of this study was to ascertain the understanding of colorectal cancer symptoms and perception of screening in a representative sample of patients served by the surgical department of Mayo General Hospital, and to examine the potential enthusiasm of this cohort to undergo colonoscopy as part of a screening program.

## Methods

### Survey

A qualitative prospective anonymous survey-based cohort study was undertaken.

The survey was adapted from that utilised by Harewood et al. [5], and sought information regarding:

•Patient demographics

•Reason for clinic attendance (i.e. colorectal symptomatology or other)

•Awareness of cancer statistics – causes of cancer death in Ireland, lifetime colorectal cancer risk

•Awareness of colorectal cancer risk factors and symptoms

•Understanding of the concept of screening

•Acceptability of colonoscopy as a screening tool

•Knowledge of Faecal Occult Blood Testing (FOBT)

•Impact of time off work and loss of earnings as a deterring factor to screening compliance

•Factors deterring patients from colonoscopy

The survey was distributed to a cohort of patients attending a general surgical outpatient clinic in Mayo General Hospital over a one-month period. Written information was provided along with the survey facilitating informed consent, which was garnered by means of ticking a box on the first page of the survey. All data was collected anonymously.

### Ethical considerations

Ethical Approval was sought from and granted by the Mayo General Hospital Research Ethics Committee following Chairperson’s review.

### Statistical analysis

Statistical analyses were performed on the data utilising PASW (version 18) software. Continuous data was assessed for normality of distribution using Shapiro-Wilk test [6], and parametric and non-parametric tests selected as appropriate.

## Results

### Demographics and presentation

The total number of surveys returned was one hundred and thirty-six, with females representing sixty-five per cent of respondents. The median age of participants was fifty-two years (range 18–87), which is significantly younger than the cohort targeted by the new screening programme. In our study group, twenty-one per cent (n = 28) of patients fit into the target age group of 60–69 years. Overall however, thirty-six per cent (n = 49) of patients were aged 55–74 years, the ultimate target cohort of the proposed screening regime (Table 1).

**Table 1:** Patient demographics and presenting complaint

**Gender N (%)**			
	**Male**	48 (35.29)	
	**Female**	88 (64.71)	
**Age (years)**			
	**<30**	17 (12.5)	
	**31-54**	55 (40.4)	
	**55-59**	8 (5.9)	
	**60-69**	28 (20.6)	
	**70-74**	13 (9.6)	
	**>74**	10 (7.4)	
	**Not disclosed**	5	
	**Male**	**Female**	**Overall**
**Mean +/- Std**	54.15 +/- 16.42	51.01 +/- 17.48	52.11 +/- 17.11
**Median (Range)**	53 (20-79)	52 (18-87)	52 (18-87)
**Presenting complaints**			
	**Male**	**Female**	**P-value (*x***^**2**^**)**
**Blood in bowel motion**	9 (18.75)	6 (6.82)	0.012
**Bright red blood per rectum**	11 (22.92)	17 (19.32)	0.341
**Bloating**	14 (29.17)	38 (43.18)	0.215
**Flatulence**	14 (29.17)	29 (32.95)	0.954
**Diarrhoea**	12 (25)	22 (25)	0.634
**Constipation**	7 (14.58)	26 (29.55)	0.091
**Weight loss**	0 (0)	8 (9.09)	0.041
**Other colorectal pathology**	1 (2.08)	0 (0)	0.145
**Non colorectal related**	17 (35.42)	23 (26.14)	0.257
**Duration of colorectal symptoms**			
	**Male n(%)**	**Female n(%)**	**Total n(%)**
**<6months**	12 (25)	17 (19.32)	29 (21.32)
**6months – 1year**	3 (6.25)	14 (15.91)	17 (12.5)
**1-2years**	6 (12.5)	6 (6.82)	12 (8.82)
**2-5years**	3 (6.25)	14 (15.91)	17 (12.5)
**5-10years**	3 (6.25)	7 (7.95)	10 (7.35)
**Missing**	12 (25)	17 (19.32)	11
**NA**	3 (6.25)	14 (15.91)	40

Forty patients were attending the surgical clinic with symptoms unrelated to colorectal disease. The remaining ninety-six patients had symptoms ranging from weight loss to long-standing flatulence. The most common presenting complaints were bloating and flatulence, followed by changes in bowel habit (constipation/diarrhoea). There were significant differences in the patterns of presenting complaints between male and female patients, with females more likely to present with weight loss (p = 0.04, *x*^2^), and men more likely to present with bleeding per rectum (p = 0.012, *x*^2^).

There were no significant differences in age of patients with suspicious symptoms compared to those without. Patients presenting with problems unrelated to colorectal disease were younger than patients presenting with lower gastrointestinal pathology, but this difference was not statistically significant.

The majority of patients presented relatively acutely, with symptoms persisting less than six months (21%). Males (25%) were more likely than females (19%) to present earlier; females were more likely to allow symptoms persist beyond one year (31%) compared to male counterparts (25%).

### Epidemiology of colorectal cancer

When patients were asked to pick the most common cause of cancer-related death in Ireland (the options to them given were lung, prostate, ovary, colon and breast), the most commonly selected answer was breast cancer (n = 50; 37%). Thirty-nine (29%) patients correctly recognised lung cancer as the most common cause of cancer death in Ireland, while thirty (22%) respondents selected colon cancer (Table 2).

**Table 2:** Patient knowledge of epidemiology, risk factors and symptoms of colorectal cancer

	**Overall**		**Target group for screening (aged 55-75years; n = 49)**
**Lifetime risk of colorectal cancer**			
**Risk**	**N(%)**		
*1 in 5*	17 (13)		5 (10)
*1 in 10*	25 (18)		8 (16)
*1 in 15*	14 (10)		6 (12)
*1 in 100*	42 (31)		14 (29)
*1 in 1000*	13 (10)		2 (4)
*Missing*	25 (18)		14 (29)
**Risk factors for colorectal cancer**			
**Factor**	**N(%)**		
*Age*	40 (29)		10 (20)
*Gender*	6 (4)		0 (0)
*Smoking*	54 (40)		17 (35)
*Stress*	64 (47)		21 (43)
*Family History*	77 (57)		28 (57)
*Alcohol*	40 (29)		9 (18)
**Symptoms concerning for colorectal cancer**			
**Symptom**	**N(%)**		**Association between having symptom and associating it with colorectal cancer (*x***^**2**^**)**
*Blood in bowel motion*	84 (62)	31 (63)	0.868
*BRBPR*	61 (45)	20 (41)	0.301
*Bloating*	15 (11)	4 (8)	0.165
*Flatulence*	9 (7)	3 (6)	0.299
*Diarrhoea*	32 (24)	10 (20)	0.232
*Constipation*	23 (17)	7 (14)	0.182
*Weight Loss*	68 (50)	22 (45)	0.649

Respondents were also asked to estimate the lifetime risk of acquisition of colorectal cancer. The majority of people incorrectly selected “1in100” (n = 42, 31%). Seventeen patients (13%) judged their risk to be as high as 20%. Fourteen (10%) correctly selected a risk of “1 in 15”.

In questions relating to respondents’ knowledge of colorectal cancer risk factors: six patients selected gender (4%). However, only forty (29%) patients correctly acknowledged age as bearing a risk, while patients more often selected factors such as stress (n = 64; 47%), alcohol consumption (n = 40; 29%) and smoking (n = 54; 40%)). The majority of people were aware of the contributory significance of family history (n = 77; 57%).

### Symptoms of colorectal cancer

In questions relating to respondents’ understanding/knowledge of colorectal cancer symptoms: eighty-four (62%) of patients correctly identified blood in the bowel motion as a worrying symptom, and sixty-one (45%) would be concerned about bright red bleeding per rectum. Nine patients (7%) incorrectly believed flatulence to be a symptom of colonic cancer. Only sixty-eight patients (50%) were concerned about weight loss and only fifty-five (40%) about altered bowel habit. There was no relationship between having a suspicious symptom (weight loss, altered bowel habit or bleeding per rectum) and the patients’ association of that symptom with cancer.

### Colonoscopy

When asked to estimate the false negative rate of colonoscopy the majority of respondents believed the incidence of false negative colonoscopy to be either 0-1% (n = 41; 30%), or 1-5% (n = 41; 30%). Eight (6%) patients believed the risk to be as high as 10-20%.

When asked to define what they felt to be an appropriate interval for re-screening in the case of a normal colonoscopy. Thirty-two per cent (n = 43) stated “1 year”, with a further twenty-five (18%) choosing “2 years”. Worryingly, ten (7%) patients would be reassured by a normal colonoscopy and would not represent for screening, while ninety-eight per cent of respondents reported that they would represent to their GP (n = 99, 73%) or the Emergency Department (n = 34, 25%) if experiencing on-going symptoms following a negative colonoscopy (Table 3).

**Table 3:** Factors deterring patients from Colonoscopy

	**Overall**	**Target Group for screening**
**Factor**	**N(%)**	
Fasting	4(3)	1 (2)
Time Off Work	3 (2)	1 (2)
Bowel preparation	45 (33)	17 (35)
Sedation	7 (5)	2 (4)
Perforation	28(21)	11 (22)
Discomfort	34 (25)	6 (12)
Embarrassment	27(20)	7 (14)

### Screening

Twenty-six (19%) patients correctly recognised “screening” as testing of an asymptomatic population, while the remainder associated the term with testing people with a positive family history (n = 56, 41%), or patients with symptoms (n = 54, 40%). There was a significant correlation between patients citing family history as a risk factor of colorectal disease and those patients selecting patients with a positive family history as the screening cohort (p = 0.002, *X*^2^).

When asked whether they would undergo screening as an asymptomatic patient in order to pick up early cancer, one hundred and two patients (75%) would comply with the screening test. Thirty-four patients would not consent to a colonoscopy in the absence of symptoms. Interestingly, only forty-seven patients (35%) were aware of FOBT, with only nine (7%) having been offered this service by their family practitioner.

Patients were then asked to define what factors they felt to be most off-putting about a colonoscopy. Twenty-eight (21%) patients were understandably concerned about the risk of perforation. Larger proportions were discouraged, however, by fear of discomfort (n = 34, 25%) or embarrassment (n = 27, 20%). Time off work was also found to be a significant deterrent. The majority of patients were willing to miss a morning (n = 23; 17%) or single day (n = 49; 36%), while 3 (2%) patients felt it completely unacceptable to take time off. Thirteen (10%) people however, were willing to take up to 3 days off.

### Responses in the target screening population

Forty-nine respondents of all those surveyed were aged between 55 and 74 years of age, and represent therefore those patients soon to be targeted for screening, including 32 (65%) females and 17 (35%) male patients. Thirty-two patients attended the clinic with symptoms of colorectal pathology, with the remaining patients attending for another unrelated surgical complaint. Of the symptomatic patients in this age group the most commonly cited symptoms included flatulence (n = 16; 50%); bloating (n = 14; 44%) and changing bowel habit (n = 15; 47%). Nine patients (28%) presented with isolated bleeding per rectum and two (6%) further patients complained of unexplained weight loss. Twenty-one patients had symptoms on-going for longer than one year (66%).

When asked to identify symptoms worrying for colorectal cancer, compared to asymptomatic patients, symptomatic patients were more likely to be suspicious of symptoms such as bleeding per rectum (44% -v- 24%); blood in stool (66% -v- 53%); diarrhoea (19% -v- 18%), constipation (22% -v- 0%) and weight loss (44% -v- 35%). Symptomatic patients were also more inclined to be worried about less sinister symptoms such as bloating (13% -v- 0%) and flatulence (9% -v- 0%) compared to asymptomatic controls. Overall, the proportion of this cohort quoting individual symptoms as worrying did not deviate greatly from the overall sample (Table 2). Of this cohort in particular, ten patients (20%) considered increasing age to be an associated risk factor for the development of colorectal cancer.

Nineteen patients (39%) in this cohort were aware of FOBT, and four (8%) of these had been made aware of its availability by their General Practitioner (GP).

Eight (16%) of these patients correctly defined screening, while 22 (45%) believed its practice to apply only to patients with a positive family history. 26 patients (53%) of this particular cohort would accept screening in the absence of symptoms.

### Barriers to uptake of colonoscopic screening

Patients were asked to select which factors would negatively impact on their compliance with colonoscopic investigations. The most commonly cited factors overall were the need for bowel preparation (n = 45 (33%)), and discomfort (n = 34, 25%). Specifically in target group for screening however, discomfort (n = 6 (12%)) was less often cited than the risk of perforation (n = 11 (22%)), and again the need for bowel preparation was the most commonly quoted dissuasive factor (n = 17 (35%)). In patients outside of the target screening age group (median age = 45 years), the risk of discomfort was mentioned as an off-putting factor as often as the need for bowel preparation (n = 28 (34%)), followed by the risk of embarrassment (n = 20, 24%). The risk of perforation (n = 17, 21%) was the fourth most commonly cited factor in this particular cohort.

## Discussion

It is well recognised that lack of cancer awareness in the community can have deleterious effects on time to presentation and, unsurprisingly therefore, on overall survival [7-10].

There remains a real lack of understanding among the Irish patient population regarding colorectal disease. This is particularly concerning considering that this cohort of patients, taken from outpatient attendees, represents patients with positive health-seeking behaviour. The symptomatic patients in this sample were concerned enough about their symptoms to attend a clinic for surgical review, and yet in many cases did not make any association between lower gastrointestinal symptomatology and potential malignancy. Furthermore, a significant proportion of patients allowed symptoms to persist beyond one year before attending for investigation. Colorectal cancer in particular remains a topic of confusion in the community, with large scale population surveys having recorded especially poor awareness of colorectal cancer risk factors among the general public, as well as a reluctance to discuss lower gastrointestinal symptoms or undergo colorectal screening [5,11-18].

It is interesting to note that, in line with other studies [5], our cohort demonstrated a particular concern for Breast cancer as a cause of cancer death. The highly successful “Pink Ribbon” campaign and the introduction of “Breast Cancer Awareness Month” annually every October has led to dramatic increase in motivation for patients to attend for screening, and have therefore resulted in an increased rate of detection of breast cancers [5]. This represents how powerful public health initiatives can be in influencing health-seeking behaviour. It reflects also an inherent willingness of patients to comply with screening. This compliance has been exemplified previously in an Irish Cohort [19]. This type of strategy has been applied to other pathologies including oesophageal cancer (Lollipop Day) and Lung Cancer (November as Lung Cancer Awareness month), and is imminently applicable to colorectal disease also.

This study highlights a particularly interesting finding. A significant proportion of patients presented to the outpatient clinic with symptoms suggestive of colorectal pathology. There was still a failure in these patients however to recognise these symptoms as concerning for colorectal cancer. Comparing symptomatic and asymptomatic patients, symptomatic patients were more likely to believe colorectal symptoms quoted to be sinister, although this was not statistically significant. Even in the symptomatic patient however, worrying symptoms such as bleeding per rectum and weight loss were underestimated in their significance. This reflects a poverty of awareness of colorectal cancer symptoms, as well as underestimation of the risk of colorectal cancer in the general community. Indeed, forty per cent of patients underestimated their risk when directly questioned on this point, with a further eighteen per cent failing to select an answer.

Patients also displayed no clear understanding of risk factors for colorectal cancer, which may deter asymptomatic patients from attending for screening. There was a poverty of recognition of increasing age as a risk factor, especially in the age group most likely to be targeted for screening (10(20%) –v- 28(34%)). Fifty-seven per cent of patients acknowledged the contribution of family history in the risk of this disease. Our results compare favourably to those of Harewood et al [5] in 2009, where only thirty-one per cent of respondents were aware of the influence of family history and only nine per cent of the impact of increasing age on risk. This may reflect increased knowledge as result of enhanced public education initiatives over the last four years in advance of the formal introduction of screening. Further efforts are needed however to enhance improvements in the public understanding of this disease.

It is clearly obvious that not only is knowledge of colorectal cancer in the community poor, but opportunistic intervention and education by GPs is limited. Of the forty-nine patients of our sample fulfilling the age criterion for screening, only four (8%) had received any information regarding screening or FOBT from their GP.

Previous studies have identified paucity of knowledge of colorectal cancers even in the symptomatic cohort [4]. Increased efforts are needed to educate patient awareness therefore, to increase compliance with the screening program, but also to encourage symptomatic patients to present at an earlier, and more salvageable, stage of disease.

In the current unease of the Irish economic situation, we were particularly interested to see whether loss of earnings resulting from time off work would impact on patients’ compliance with the screening process. The majority of patients were willing to miss half a day or one full day (53%, n = 72), with stepwise decreases in proportions of patients willing to miss any further increments in loss of working days. This is a particularly relevant factor in colonoscopic screening, as the process involves not only the test itself, but also bowel preparation on the day prior to screening, and a period of recovery following sedation. Compared to mammography or other screening initiatives, it seems arduous and unsurprisingly dissuasive.

This study clearly exemplifies the deficiencies in public awareness of colorectal cancer symptoms and risks, and highlights the need for augmentation of public health initiatives.

The single-centre nature of this study is a limiting factor but the findings are in keeping with previously published work from other centres. The majority of previous work in Ireland has focused on urban-dwellers attending tertiary specialist centres. We believe this study to be the first in Ireland to examine the attitudes and awareness of a rural based population attending a general peripheral centre. In rural Ireland, patients are more dependent on primary care and the paucity of education offered by GPs to this sample is concerning. It would be interesting to repeat the study in a sample from patients in the community rather than in the health-seeking population attending for review.

## Conclusion

This study clearly demonstrates a lack of public insight into signs and symptoms of colorectal cancer. The sample included in this study included a wide variety of ages, gender and symptom profile, from a predominantly rural population. It therefore provides a valid representation of the health-seeking population in the West of Ireland. An urgent requirement exists for increased Public Health initiatives to increase knowledge in the community, to encourage family practitioners to educate patients and offer them faecal occult blood testing (FOBT) when indicated, and to ensure compliance with the screening program when it is initiated. A particularly interesting and novel finding in this study is the appearance of loss of time and earnings as a deterrent in screening participation. This is particularly valid in the current economic climate, and should be taken into account in patient education initiatives, or indeed in the planning of colonoscopy lists.

## Competing interest

Nil to Disclose

## Authors’ contributions

Design and Concept: Waldron RM, McVeigh T. Data Collection: McVeigh T, Waldron RM, Barry K, Mahmood A. Data Analysis: McVeigh T, Lowery AJ Manuscript Preparation: McVeigh T, Lowery AJ, Waldron RM. Manuscript Critical Appraisal: Lowery AJ, Waldron RM, Barry K, Mahmood A. All authors read and approved the final manuscript.

## Pre-publication history

The pre-publication history for this paper can be accessed here:

http://www.biomedcentral.com/1471-2482/13/20/prepub

## References

[B1] Cancer in Ireland 2011 Statistical Report of The National Cancer Registry of Ireland 2011

[B2] Wilson JMG Jungner G Principles and practice of screening for disease 1968 Geneva: World Health Organization

[B3] Update on Progress on the Implementation of the National Colorectal Cancer Screening Programme from http://www.cancerscreening.ie/colorectal.html

[B4] Manning A Waldron R Barry K Poor awareness of colorectal cancer symptoms; a preventable cause of emergency and late stage presentation Ir J Med Sci 2006 175 4 55 57 10.1007/BF03167968 17312830

[B5] Harewood GC Murray F Patchett S Garcia L Leong WL Lim YT Assessment of colorectal cancer knowledge and patient attitudes towards screening: Is Ireland ready to embrace colon cancer screening? Ir J Med Sci 2009 178 1 7 12 10.1007/s11845-008-0163-x 18584273

[B6] Shapiro SS An analysis of variance test for normality (complete samples) 1964

[B7] Mitchell E Macdonald S Campbell NC Weller D Macleod U Influences on pre-hospital delay in the diagnosis of colorectal cancer: A systematic review Br J Cancer 2008 98 1 60 70 10.1038/sj.bjc.6604096 18059401 PMC2359711

[B8] Macdonald S Macleod U Campbell NC Weller D Mitchell E Systematic review of factors influencing patient and practitioner delay in diagnosis of upper gastrointestinal cancer Br J Cancer 2006 94 9 1272 1280 10.1038/sj.bjc.6603089 16622459 PMC2361411

[B9] MacLeod U Mitchell ED Burgess C MacDonald S Ramirez AJ Risk factors for delayed presentation and referral of symptomatic cancer: Evidence for common cancers Br J Cancer 2009 101 SUPPL. 2 S92 S101 19956172 10.1038/sj.bjc.6605398 PMC2790698

[B10] Richards MA Westcombe AM Love SB Littlejohns P Ramirez AJ Influence of delay on survival in patients with breast cancer: A systematic review Lancet 1999 353 9159 1119 1126 10.1016/S0140-6736(99)02143-1 10209974

[B11] Keighley MRB O'Morain C Giacosa A Ashorn M Burroughs A Crespi M Public awareness of risk factors and screening for colorectal cancer in Europe Eur J Cancer Prev 2004 13 4 257 262 10.1097/01.cej.0000136575.01493.9b 15554552

[B12] Domati F Travlos E Cirilli C Rossi G Benatti P Marino M Attitude of the Italian general population towards prevention and screening of the most common tumors, with special emphasis on colorectal malignancies Intern Emerg Med 2009 4 3 213 220 10.1007/s11739-008-0184-5 18807148

[B13] Gimeno-García AZ Quintero E Nicolás-Pérez D Jiménez-Sosa A Public awareness of colorectal cancer and screening in a Spanish population Public Health 2011 125 9 609 615 10.1016/j.puhe.2011.03.014 21794885

[B14] Power E Miles A Von Wagner C Robb K Wardle J Uptake of colorectal cancer screening: System, provider and individual factors and strategies to improve participation Future Oncol 2009 5 9 1371 1388 10.2217/fon.09.134 19903066

[B15] Aubin-Auger I Mercier A Lebeau JP Baumann L Peremans L Van royen P Obstacles to colorectal screening in general practice: A qualitative study of GPs and patients Fam Pract 2011 28 6 670 676 10.1093/fampra/cmr020 21551256

[B16] Jones RM Woolf SH Cunningham TD Johnson RE Krist AH Rothemich SF The Relative Importance of Patient-Reported Barriers to Colorectal Cancer Screening Am J Prev Med 2010 38 5 499 507 10.1016/j.amepre.2010.01.020 20347555 PMC2946819

[B17] McLachlan SA Clements A Austoker J Patients' experiences and reported barriers to colonoscopy in the screening context-A systematic review of the literature Patient Educ Couns 2012 86 2 137 146 10.1016/j.pec.2011.04.010 21640543

[B18] Ramos M Llagostera M Esteva M Cabeza E Cantero X Segarra M Knowledge and attitudes of primary healthcare patients regarding population-based screening for colorectal cancer BMC Cancer 2011 11 art. no. 408 10.1186/1471-2407-11-408 PMC3190390 21942990

[B19] Codd MB Mortality from breast cancer in Ireland prior to the introduction of population-based mammographic screening Ir J Med Sci 1999 168 2 87 92 10.1007/BF02946471 10422384

